# Promoter Hypomethylation of Maspin Inhibits Migration and Invasion of Extravillous Trophoblast Cells during Placentation

**DOI:** 10.1371/journal.pone.0135359

**Published:** 2015-08-11

**Authors:** Xinwei Shi, Hao Liu, Jing Cao, Qing Liu, Guiju Tang, Wanlu Liu, Haiyi Liu, Dongrui Deng, Fuyuan Qiao, Yuanyuan Wu

**Affiliations:** 1 Department of Obstetrics and Gynecology, Tongji Hospital, Tongji Medical College, Huazhong University of Science and Technology, Wuhan, Hubei, China; 2 Department of Urology, Wuhan Third Hospital, guanggu on campus, Wuhan, Hubei, China; 3 Department of Obstetrics and Gynecology, Union Hospital, Tongji Medical College, Huazhong University of Science and Technology, Wuhan, Hubei, China; Georgia Regents University, UNITED STATES

## Abstract

**Objective:**

Extravillous trophoblast (EVT) cells invade the endometrium and the maternal spiral arterioles during the first trimester. Mammary Serine Protease Inhibitor (Maspin, SERPINB5) plays a putative role in regulating the invasive activity of cytotrophoblasts. The maspin gene is silenced in various cancers by an epigenetic mechanism that involves aberrant cytosine methylation. We investigated the effect of the methylation status of the maspin promoter on the maspin expression and the aggressiveness of EVT cells.

**Methods:**

Western blotting was used to detect the maspin protein expression in EVT cells upon hypoxia. The proliferative ability, the apoptosis rate and the migration and invasiveness were measured with Cell Counting Kit-8 assay, Flow Cytometry technology and Transwell methods. Subsequently, we treated cells with recombinant maspin protein. The methylation degree of maspin promoter region upon hypoxia/ decitabine was detected by bisulfite sequencing PCR and methylation-specific PCR. Finally, we explored the effects of decitabine on maspin protein expression and the aggressiveness of EVT cells.

**Results:**

Hypoxia effectively increased maspin protein expression in EVT cells and significantly inhibited their aggressiveness. The addition of recombinant maspin protein inhibited this aggressiveness. Decitabine reduced the methylation in the maspin promoter region and effectively increased the maspin protein expression, which significantly weakened the migration and invasiveness of EVT cells.

**Discussion:**

The methylation status of the maspin promoter is an important factor that affects the migration and invasion of EVT cells during early pregnancy. A decrease in the methylation status can inhibit the migration and invasion of EVT cells to affect placentation and can result in the ischemia and hypoxia of placenta.

## Introduction

The differentiation of the blastocyst trophectoderm results in multiple trophoblastic cell lineages that have unique biological activities[1]. These lineages are responsible for the evolution of the human placenta. Extravillous trophoblast (EVT) cells, which have tumor-like properties and are under critical control, invade the endometrium and the maternal spiral arterioles during the first trimester. Failure to achieve this invasion can lead to shallow placenta implantation and result in placenta defect diseases, such as preeclampsia and fetal growth restriction[2]. Many studies have found that the EVT invasion process primarily depends on the oxygen levels[3].

Mammary Serine Protease Inhibitor (Maspin, SERPINB5) was identified in normal mammary epithelia and invasive mammary cancer cells[4, 5]. It is down-regulated in many types of cancer, such as breast cancer[6] and prostate cancer[7]. The rephrased of maspin inhibits growth, cell migration and invasion and increases cell adhesion and apoptosis[8–10]. The expression of maspin is maximized in the term placenta compared to the first and second trimester tissues, and cytotrophoblasts isolated from the term placenta correspondingly are least invasive compared to first and second trimester cytotrophoblasts[11]. The addition of recombinant maspin protein significantly decreased cytotrophoblast invasion in vitro[11]. In our previous research, we also found that maspin expression was increased at the mRNA and protein levels in preeclamptic placental tissues compared to nonpreeclamptic group[12], the mRNA expression of maspin in human First-Trimester Extravillous Trophoblast Cell Line (TEV-1) in chemical hypoxic environment, which was induced by chemical reagent (CoCl_2_), was significantly increased and CoCl_2_ inhibited the proliferative ability and the migrative ability of TEV-1 cells[13]. Thus, it is speculated that maspin plays a putative role in regulating the invasive activity of cytotrophoblasts. The down-regulation of maspin expression may be critical at the time of implantation and early placental development.

Many cancer studies[14–18] showed that an epigenetic mechanism that involves aberrant cytosine methylation silences the maspin gene, which revealed that DNA methylation plays an important role in regulating the expression of maspin. The maspin gene promoter is hypomethylated in placental tissues and densely methylated in maternal blood cells; the maternal plasma concentration of unmethylated maspin sequences was significantly increased in preeclamptic pregnancies compared with nonpreeclamptic pregnancies[19]. In our previous research, we found that the methylation level of the maspin promoter was significantly hypomethylated in preeclamptic placentas compared to nonpreeclamptic placentas[12] and the demethylating reagent (5-Aza-2’-deoxycytidine) increased mRNA expression of maspin in TEV-1 and inhibited the migrative ability of TEV-1[13]. Thus, a change in the methylation status of the maspin promoter may participate in the pathogenesis of shallow trophoblast invasion.

Based on the above descriptions and research, we speculate that maspin, which is under the control of the methylation degrees of maspin promoter regions, plays an important role in regulating the biological functions of trophoblast cells. To test this hypothesis, we investigated the effect of hypoxia on the protein expression of maspin and biological functions of EVT cells; EVT cells were used as a model to study the pathogenesis of shallow placenta implantation. We treated these cells with recombinant maspin protein (r.M) and observed the effect on the biological functions of EVT cells. Furthermore, the effects of decitabine, a demethylation reagent, on the methylation status of the maspin promoter, protein expression of maspin and biological functions of EVT cells were analyzed.

## Materials and Methods

### 1 TEV-1 cell line culture

The TEV-1 was used as a model to study the process of placentation. This cell line[20] was graciously provided by Doctor S.W. Tsao from the University of Hong Kong and maintained in DMEM/F-12 (1:1) media (HyClone) supplemented with 10 percent fetal calf serum (Gibco). The cells were incubated in a humidified atmosphere with 5% CO_2_ at 37°C.

### 2 Reagent intervention

The TEV-1 cells were treated with 500nmol/L 5-aza-2’-deoxycytidine (decitabine, DI)(Sigma)[21], 300μmol/L CoCl_2_(Sigma)[22], 100ng/mL recombinant maspin protein (Pepretech) or PBS control at 0h and cultured for 12h, 24h, 48h or 72h at 37°C/5% CO_2_. The cells were harvested for downstream DNA and protein applications.

### 3 DNA extraction and sulfitation

DNA was extracted from cultured cells using DNA Extraction Kit (TianGen, Beijing, China) and bisulfite-converted using the EZ DNA Methylation Kit (Zymo Research, Orange, CA, USA) according to the manufacturer’s protocol.

### 4 Bisulfite pyrosequencing and Methylation-specific PCR

To investigate the methylation status of the maspin promoter region, 13 CpG sites in the CpG island of the maspin promoter region were amplified using the bisulfite sequencing PCR (BSP) of the bisulfite-treated DNA. The 4 groups of TEV-1 cells were sequenced. Methylation-specific PCR (MSP) was then performed with methylated and unmethylated maspin PCR primers to determine the methylation status of maspin. The primers used for MSP and the conditions of PCR can be found elsewhere[12].

### 5 Western blot analysis

Protein was extracted using a lysis buffer and protease inhibitor cocktail (Thermo Scientific). After centrifugation, the supernatant was used for a western blot assay (maspin monoclonal antibody (BD Pharmingen, San Diego, USA, dilution 1:500), glyceraldehyde phosphate dehydrogenase (GAPDH) antibody (Santa Cruz, California, USA, dilution 1:1000)).

### 6 Cell Proliferation Capacity Assay with the Cell Counting Kit-8

TEV-1 cells were seeded into 96-well plates (1×10^4^/well) and incubated in 100 μL of culture medium supplemented with 10% FBS and various reagents (PBS, CoCl_2_, and 5-aza) for 6, 12, 24 and 48 h. After incubation, we determined the cell proliferation using the Cell Counting Kit-8 (CCK8). The absorbance at 450 nm was measured to determine the number of viable cells in each well. All procedures were performed in triplicate.

### 7 Apoptosis Assay

TEV-1 cells were seeded into 6-well plates (5×10^4^/well) and incubated in 3 mL of culture medium supplemented with 10% FBS and various reagents (PBS, CoCl_2_, and 5-aza) for 6, 12, 24, and 48 h. After incubation, we determined the level of cell apoptosis using the Annexin V-PI double dye apoptosis kit and FAC Sort flow cytometry (FCM) instrument (Becton Dickinson, USA). All procedures were performed in triplicate.

### 8 Migration and Invasion Ability Assay by Transwell

Cells (5×10^4^/ml) in serum free medium, which contained intervention reagent, were transferred to the upper chamber, while 600 μL of DMEM/F-12 (1:1) media supplemented with 10 percent fetal calf serum was added to the lower chamber. After incubating the cells at 37°C for 24h, the medium was removed. The wipe method was used to gently remove cells on the upper side of the filter with a cotton swab. The cells that had migrated to the lower surface were then fixed with 4% paraformaldehyde and stained with crystal violet. The average number of migrated cells was determined by counting the cells in 5 random fields (×200). Triplicate wells were analyzed in each experiment, and the experiment was repeated. The invasion assay was performed in a similar manner, but the filters were coated with 40μL of Matrigel (dilution 1:8), and the cells were incubated for 48h. Three independent experiments were performed for each assay.

### 9 Statistical Analysis

The data are presented as the mean ± standard deviation (SD). The differences among groups were assessed with a one-way analysis of variance (ANOVA) using the SPSS 17.0 package. P<0.05 was considered significant.

## Results

### 1 Hypoxia effectively increased protein expression of maspin and significantly inhibited the aggressiveness of EVT cells

**Fig 1:**
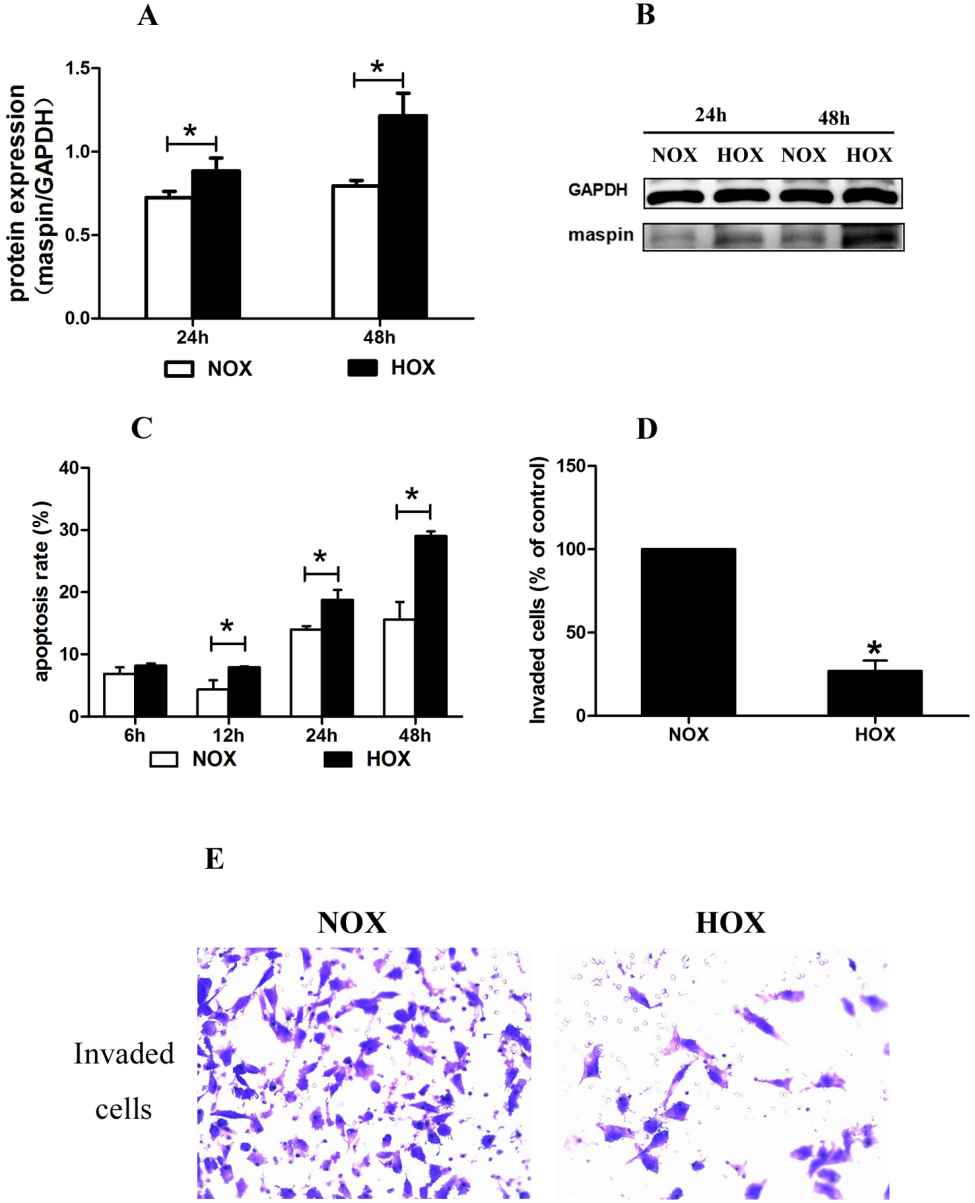
Effect of hypoxia on protein expression of maspin, apoptotic and invasive ability in TEV-1 A: The maspin protein in the normoxic and hypoxic groups expressed as a relative measure compared to GAPDH and normalized to a control sample run on each gel at two different culture time points (24h, 48h). The protein expression of maspin in the HOX group was significantly higher compared to the NOX group at both 24h and 48h, the protein expression of maspin at 48h was significantly higher compared to 24h; (*P < 0.05). B: Representative Western blot analysis of maspin protein expression in the NOX and HOX groups; C: Apoptosis rate of cells was measured by flow cytometry. The apoptosis rate in the HOX group was significantly higher compared to the NOX group at 12h, 24h and 48h. (*P < 0.05) D: The invasive ability of cells were tested by transwell method. The invaded cells in the HOX group was significantly fewer compared to the NOX group at 48h. (*P < 0.05) E: Photo of invaded cells in the NOX and HOX group (200X).

In our previous study, we found that hypoxia, which was induced by CoCl2, significantly increased the mRNA expression of maspin in EVT cells. CoCl_2_ has been used as a chemical reagent that induces a biochemical and molecular response similar to that observed under hypoxic conditions[23, 24]. In previous researches of our laboratory[13, 22, 25], we used CoCl_2_ treatment to construct hypoxic model in vitro for studying the aggressive ability of trophoblast cells and our results were consistent with many related publications[3, 26–27], which used hypoxia incubator to construct hypoxic environment. So we investigate the effect of the hypoxia-mimetic CoCl_2_ in subsequent study. To investigate the potential effects of hypoxia on the protein expression of maspin in TEV-1 cells, the cells were exposed to hypoxic (HOX) conditions for various times. Western blot analysis were used to assess changes in protein expression. Compared with normoxic (NOX) trophoblast cells, the protein expression of maspin was also significantly increased in hypoxic trophoblast cells (1.22- and 1.53-fold at 24h and 48h, respectively) [Fig 1].

It was also found that hypoxia significantly inhibited the proliferative ability and the migrative ability of EVT cells in our previous study. And in this study, we examined the apoptosis and invasion of trophoblast cells upon hypoxia. Hypoxia significantly increased the apoptosis rate of trophoblast cells by 1.82-, 1.34- and 1.86-fold at 12h, 24h and 48h, respectively. At 48h, the number of EVT cells in HOX was 78.0% of that in NOX, and the apoptosis rates in NOX and HOX were respectively 15.63±2.81(%) and 29.01±0.76(%). But the number of invasive cells in HOX at 48h was 26.93% of that in NOX. Thus not only less number of EVTs but also the weakening of invasive ability in HOX lead to decreased number of invading EVTs. [Fig 1].

### 2 Recombinant maspin protein significantly inhibited the aggressiveness of EVT cells

**Fig 2:**
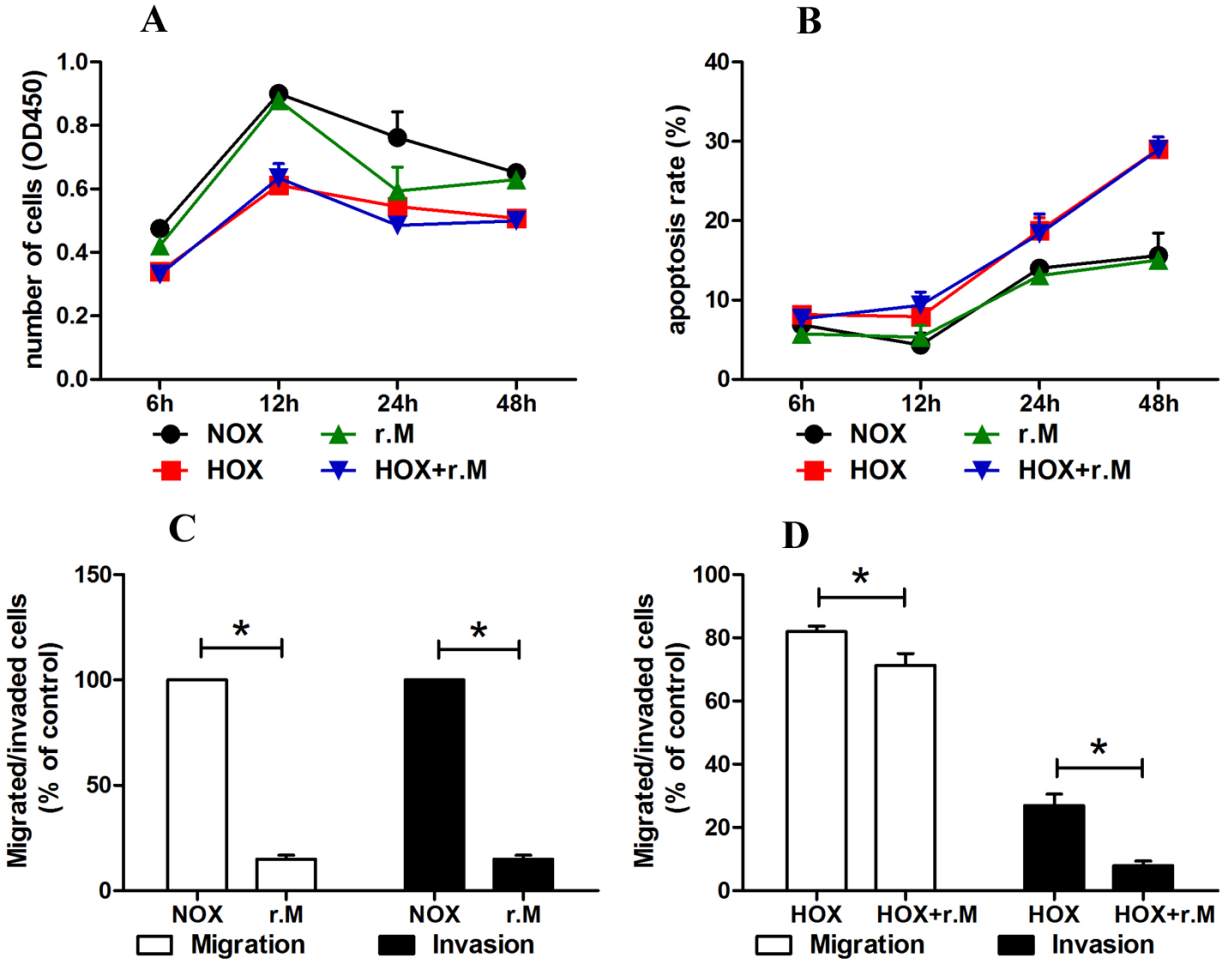
Effect of recombinant maspin protein on proliferative, apoptotic, migrative and invasive ability in TEV-1 A: The number of cells in NOX, HOX, r.M and HOX+r.M groups was measured by ELISA. Recombinant maspin protein significantly weakened the proliferative ability of TEV-1 cells in NOX group at 24h, but overall it had no significant effect on the proliferative ability of TEV-1 cells in either NOX or HOX group. B: The apoptosis rate of cells in NOX, HOX, r.M and HOX+r.M groups was detected by FCM. Recombinant maspin protein had no significant effect on the apoptosis rate of TEV-1 cells in either NOX or HOX group. C: The migrated/invaded cells were counted to estimate the effect of recombinant maspin protein on normoxic EVT cells. Recombinant maspin significantly weakened the migrative ability and the invasive ability of TEV-1 cells in the normoxic EVT cells. (*P < 0.05) D: The migrated/invaded cells were counted to estimate the effect of recombinant maspin protein on hypoxic EVT cells. Recombinant maspin significantly weakened the migrative ability and the invasive ability of TEV-1 cells in the hypoxic EVT cells. (*P < 0.05).

To examine the potential effects of recombinant maspin protein on the aggressiveness of trophoblast cells, the cells were treated with recombinant maspin protein at various times. Recombinant maspin protein significantly weakened the proliferation of TEV-1 cells in the NOX group at 24h but did not significantly affect the overall proliferation of TEV-1 cells in either the NOX or HOX group. Recombinant maspin protein also did not significantly affect the apoptosis rate in either the NOX group or HOX group at 6h, 12h, 24h and 48h. Nevertheless, it significantly weakened the migration (0.861- and 0.870-fold) and invasion (0.149- and 0.295-fold) of TEV-1 cells in the NOX and HOX groups [Fig 2].

### 3 Hypoxia/DI significantly reduced the methylation ratios at the CpG island in the maspin promoter region of EVT cells

**Fig 3:**
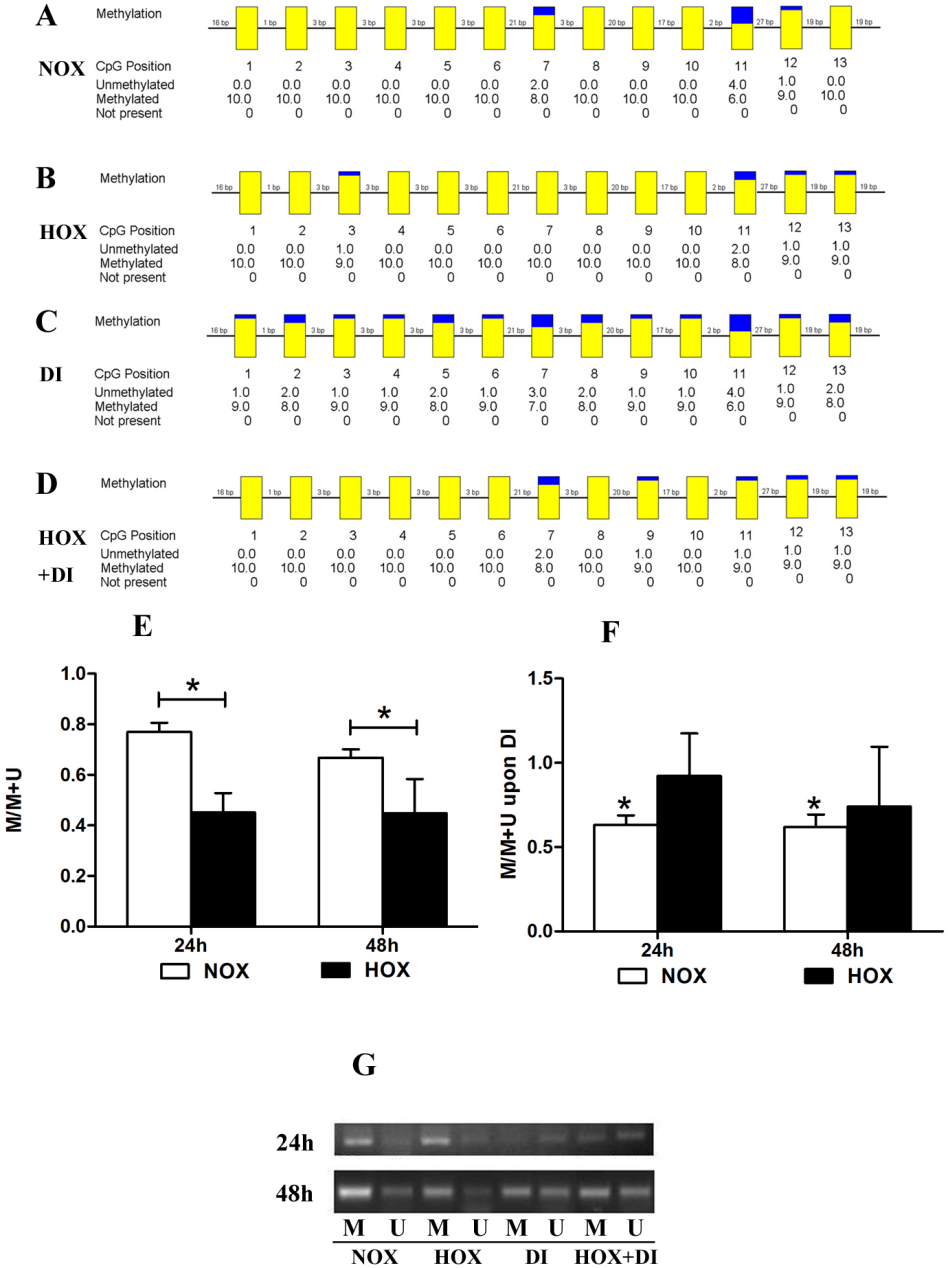
Status of methylation at the CpG site in the maspin promoter region of TEV-1 cells upon hypoxia and DI A-D: Bisulfite sequencing revealed a methylated CpG island in the maspin promoter region of TEV-1 cells in four groups. Each square represents an individual CpG site, and the sizes of the yellow parts indicate fractional methylation. The yellow parts represent methylation-positive results, and the blue parts represent methylation-negative results. E: The extent of methylation at the CpG site was calculated using the following formula: [extent of methylation/(extent of methylation + extent of unmethylation)]. The methylation rates of the maspin promoter in the HOX group were significantly decreased compared to the NOX group at 24h and 48h. (*P < 0.05). F: DI significantly reduced the methylation rates of the maspin promoter in the NOX group, but it had no significant effect on the methylation rates of the maspin promoter in the HOX group. (*P < 0.05). G: MSP analysis of maspin promoter in TEV-1 cells of four groups. All the 4 groups show the presence of both unmethylated and methylated DNA in the placental tissues, respectively. The bands detected by the methylated primer represent methylated maspin (M), and the bands detected by the unmethylated primer represent unmethylated maspin (U).

We used methylation-specific PCR on bisulfite-treated DNA to reveal the methylated CpG island in the maspin promoter region of trophoblast cells upon hypoxia or/and DI. We also used MSP to detect the extent of methylation at the CpG island. Hypoxia significantly decreased the methylation ratios of the maspin promoter (0.586- and 0.671-fold at 24h and 48h, respectively). Decitabine significantly reduced the methylation ratios of the maspin promoter in normoxic trophoblast cells. There was no significant difference of MASPIN promoter methylation levels between hypoxia versus DI treatments, which might result from the big standard deviation. [Fig 3].

### 4 DI significantly increased the protein expression of maspin, and effectively inhibited the aggressiveness of EVT cells

**Fig 4:**
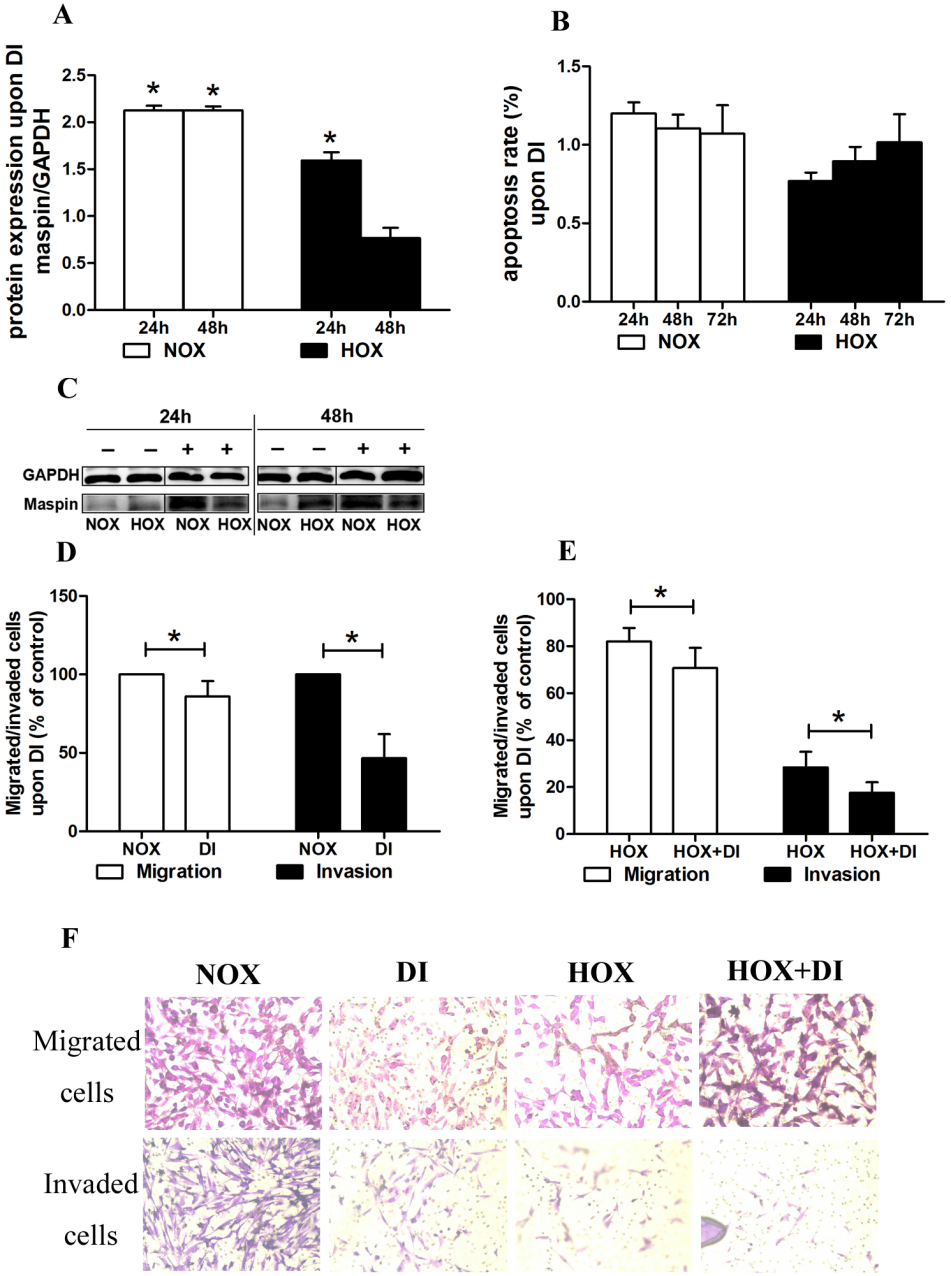
Effect of DI on maspin expression, aggressive ability of TEV-1 cells. A: Representative Western blot analysis of maspin protein expression. DI increased the protein expression of maspin in TEV-1 cells in the NOX group at 24h and 48h. DI increased the protein expression of maspin in TEV-1 cells in the HOX group at 24h, but had no significant effect on the protein expression of maspin in TEV-1 cells in the HOX group at 48h. (*P < 0.05). B: We investigated the effect of decitabine on the apoptosis rate of the normoxic/hypoxic EVT cells. DI had no significant effect on the apoptosis rate of TEV-1 cells in the NOX and HOX group. C: Representative Western blot analysis of maspin protein expression in the NOX and HOX groups upon DI at two different culture time points (24h, 48h). (+: with DI;-: without DI). D: The migrated/invaded cells were counted to estimate the effect of decitabine on normoxic EVT cells. Decitabine significantly weakened the migrative ability and the invasive ability of TEV-1 cells in the normoxic EVT cells. (The control was the NOX group. *P < 0.05) E: The migrated/invaded cells were counted to estimate the effect of decitabine on hypoxic EVT cells. Decitabine significantly weakened the migrative ability and the invasive ability of TEV-1 cells in the hypoxic EVT cells. (The control was the NOX group. *P < 0.05) F: Photo of migrated/invaded cells in the NOX, DI, HOX and HOX+DI group (200X).

In our previous study, DI increased the mRNA expression of maspin in TEV-1 cells in the NOX group and had no significant effect on the proliferation of TEV-1 cells. In this study, DI increased the protein expression of maspin in TEV-1 cells in the NOX group at 24h and 48h. DI increased the protein expression of maspin in TEV-1 cells in the HOX group at 24h but did not significantly affect the protein expression of maspin in TEV-1 cells in the HOX group at 48h. DI did not significantly affect the apoptosis ratio of TEV-1 cells in the NOX and HOX groups. Decitabine significantly weakened the migration and invasion of trophoblast cells [Fig 4].

## Discussion

The placenta is recognized as crucial in supporting the growth and development of the mammalian fetus. Extravillous trophoblast (EVT) cells, which invade the decidua and erode local spiral arteries during the first trimester, are recognized as important in the progress of placentation. If trophoblast migration and the invasiveness of EVT cells are disturbed, placenta defect diseases, such as preeclampsia and fetal growth restriction, occur. The extravillous trophoblast invasion process primarily depends on the oxygen levels[28, 29]. During early pregnancy, trophoblast differentiation takes place in a physiologically hypoxic environment, which is important for normal embryonic and placental development. At approximately 10–12 weeks of gestation, the increased O_2_ pressure is accompanied by maximum trophoblast invasion. Insufficient trophoblast invasion results in chronic placental hypoxia later in pregnancy, which leads to preeclampsia[3].

Maspin, which is a member of the serpin superfamily of protease inhibitors, suppresses several tumor types by inhibiting cell proliferation, differentiation, angiogenesis[30, 31], adhesion[10], migration[32, 33] and invasion and by promoting apoptosis[9, 34]. Hong SN et al.[35] found that maspin inhibited the invasiveness of pancreatic ductal adenocarcinoma cells. Suppressing maspin expression with snail protein enhanced the migration and invasion human prostate cancer cells[36]. Sood AK et al.[37] found that the transfection of maspin in vitro decreased the invasiveness of ovarian carcinoma cells. Dokras et al.[11] reported a negative correlation between the expression of maspin and the invasiveness of cytotrophoblast cells during human placental development. Previously published works from our laboratory revealed that the mRNA and protein expression levels of maspin increased 1.25- and 3-fold, respectively, in preeclamptic placentas compared with normal placentas[12]. Li HW et al.[38] found that the expression of maspin in gestational trophoblastic disease, in which the aggressiveness of trophoblast cells is enhanced, was significantly decreased, as evidenced by immunohistochemistry and RT-PCR assays. Thus, we speculated that maspin plays an essential role in regulating the ability of trophoblast cells. We used quantitative real-time PCR and western blot assays to detect a significant increase in the mRNA[13] and protein expression levels of maspin in EVT cells in response to hypoxia, and we found that hypoxia inhibited the proliferation[13], migration and invasion of EVT cells and promoted their apoptosis. Taken together, these data indicate a strong correlation between the expression of maspin in EVT cells and their aggressiveness. To verify this correlation, we treated EVT cells with recombinant maspin and observed a significant decrease in their invasiveness. The above results revealed that hypoxia up-regulated the expression of maspin, which decreases the aggressiveness of EVT cells.

DNA methylation is the most commonly studied epigenetic mechanism. Domann FE et al.[39] compared HMECs with 9 types of human breast cancer cell lines and found that the expression of maspin was closely related to the methylation status of the maspin promotor region. Chim SS et al.[19] reported that the concentrations of unmethylated maspin sequences in the maternal plasma are elevated by a median of 5.7-fold in preeclamptic pregnancies compared to healthy pregnancy controls. In our previous study, we found that the mean methylation level of the maspin promoter region was significantly hypomethylated in preeclamptic placentas compared to control placentas[12]. In this study, the methylation status of maspin in EVT cells was detected by BSP and MSP assays; we found that hypoxia significantly decreased the methylation rates of the maspin promoter and significantly reduced the methylation rates of the maspin promoter in normal trophoblast cells, which was in accordance with the up-regulation of maspin expression by hypoxia. The results obtained from our in vitro experiments on EVT cells also agree with studies of the placenta.

Various drugs, such as the DNA methyltransferase inhibitor, 5-aza-2’-deoxycytidine (5-Aza-CdR, decitabine, DI), can reverse the epigenetic silencing of a gene to reactivate of gene expression. Decitabine is a well-known demethylating agent used in cancer therapy. Decitabine has been shown to induce the expression of maspin in many cancer studies[14, 16–18, 40, 41], and this expression inhibits angiogenesis and invasion. In this study, DI significantly reduced the methylation rates of the maspin promoter in normal trophoblast cells according to BSP and MSP assays. Furthermore, we found that decitabine up-regulated the expression of maspin in EVT cells. Thus, the down-regulation of the maspin promoter methylation levels corresponded to an elevated expression of maspin in hypoxic or demethylated EVT cells. Additionally, decitabine also inhibited the aggressiveness (migration and invasion) of EVT cells. Thus, decitabine can be inferred to inhibit the aggressiveness of trophoblasts by reducing the methylation rates of the maspin promoter, which increases the expression of maspin.

In summary, our study showed that hypoxia effectively increased the expression of maspin and significantly inhibited the aggressiveness of EVT cells; recombinant maspin protein significantly weakened the migration and invasion of EVT cells; decitabine significantly reduced the methylation rates of the maspin promoter in normoxic EVT cells, but it effectively increased the maspin expression and significantly weakened the migration and invasion of EVT cells. These results reveal that the methylation status of the maspin promoter is an important factor that affects the migration and invasion of EVT cells during early pregnancy. A decrease in the methylation status can inhibit the migration and invasion of EVT cells to affect placentation and result in the ischemia and hypoxia of placenta, which leads to placenta insufficiency diseases, such as preeclampsia.
